# Seasonal Changes in Soil Microbial Community and Co-Occurrence Network of Species of the Genus *Corylus*

**DOI:** 10.3390/microorganisms9112228

**Published:** 2021-10-26

**Authors:** Wenxu Ma, Zhen Yang, Lisong Liang, Qinghua Ma, Guixi Wang, Tiantian Zhao

**Affiliations:** 1 State Key Laboratory of Tree Genetics and Breeding, Beijing 100091, China; mawenxu1994@163.com (W.M.); yangzhen20051993@163.com (Z.Y.); lianglscaf@126.com (L.L.); mqhmary@sina.com (Q.M.); wanggx0114@163.com (G.W.); 2 Key Laboratory of Tree Breeding and Cultivation of the National Forestry and Grassland Administration, Research Institute of Forestry, Chinese Academy of Forestry, Beijing 100091, China; 3 Hazelnut Engineering and Technical Research Center of the State Forestry and Grassland Administration, Beijing 100091, China; 4 National Forestry and Grassland Innovation Alliance on Hazelnut, Beijing 100091, China

**Keywords:** hazelnut, microbial community, structure, function, seasonal variation, co-occurrence network analyses

## Abstract

Hazelnut is one of the four major nuts in the world and has high nutritional and economic value. This study employed Illumina sequencing of ITS rDNA and 16S rRNA genes to identify the seasonal changes in soil microbial community, the predominant environmental factors driving microbial community composition, and the differences in soil microbial composition among different species of the genus *Corylus*. We found that the soil microbial community composition of species of *Corylus* changed significantly with the change in seasons. *Corylus heterophylla* and *Corylus kweichowensis* had more ectomycorrhiza in their soil compared to *Corylus avellane*. The main factor influencing fungal community composition in soil was the available potassium, while that of bacteria was the total phosphorus content. Co-occurrence network analysis revealed that the ratio of positive interaction to negative interaction in soil of *C. heterophylla* and Ping’ou (*C. heterophylla* × *C. avellane*) was higher, while the negative interaction of soil community structure in *C. avellane* was greater. The bacterial community was more stable than the fungal community according to microbial diversity and co-occurrence network analyses. The findings of this research may facilitate improvements to the production and soil system management in hazel planting processes.

## 1. Introduction

Hazelnut, produced by a shrub or small tree of *Corylus* Linn. in the family Betulaceae, is one of the four major dried fruits in the world and was reported to have originated in southwest China in the middle Eocene (~43.6 million years ago) [1,2]. Among Chinese hazel plants, *Corylus heterophylla*, mainly distributed in northern China, has been developed and utilized, while *Corylus kweichowensis*, predominantly distributed in southern China, has important potential utilization value [3]. *Corylus avellane* was introduced in China at the end of the 19th century, and a new hybrid hazel germplasm named Ping’ou which was hybridized by *C. heterophylla* and *C. avellane* had the advantages of strong resistance, high yield, and large fruit was obtained in the 1980s [4,5].

Soil biological properties and soil microbial composition change with the seasons [6,7], potentially in relation to seasonal differences in soil temperature, moisture, and soil organic matter content or autecological dynamics [8,9,10]. Seasonal environmental variables, photosynthesis, root exudates, and litter can significantly change the composition of soil microbial communities [11,12]. Previous studies showed that as the seasons changed, there was no significant change in β-glucosidase, urease, and acid phosphatase activities in the soil of a hazelnut orchard [13]. However, seasonal changes in the soil microbial community structure of *Corylus* have not yet been elucidated, and the environmental factors driving changes in the soil microbial community of *Corylus* have not been studied to date. Clarifying the seasonal dynamics of the soil microbial community of *Corylus* will facilitate the understanding of the composition of the soil microbial community of *Corylus*.

Vegetation type is the predominant factor influencing the construction of soil microbial communities [14,15,16]. The microbial community in topsoil is directly affected by vegetation types, because the difference in decomposability of litter produced by different tree species affects the abundance of microorganisms [10]. Root exudates of different types of plants may also affect the composition of soil microbial communities in deep soil [17]. Furthermore, vegetation types may indirectly affect microbial composition by regulating soil physical and chemical properties, which can directly affect soil microbial community composition [18,19]. However, there are few studies on the seasonal differences in soil microorganisms of vegetation that are of the same genus, but different species.

Soil microorganisms usually form a complex interspecific network [20]. Co-occurrence network analysis is an effective method to explore the interactions between different entities in the system and has been used to study various complex ecosystems [21,22]. However, research on the seasonal changes of co-occurrence network analysis among species of the genus *Corylus* is limited; such information could reveal the differences in the microbial community network among different species of the genus *Corylus* and the influence of seasonal changes on the network.

Therefore, in this study, the soil microbes of different species of the genus *Corylus* in different seasons were sequenced, with the aims of (1) revealing the seasonal variation in the soil microbial community of species of genus *Corylus*, (2) clarifying the differences in soil microbial composition and function among different species of *Corylus*, (3) exploring the environmental factors driving changes in soil microbial community composition of *Corylus*, and (4) studying the co-occurrence network differences in the soil microbial community in different species of the genus *Corylus* and in different seasons. We confirmed the hypothesis that the diversity, species composition, and co-occurrence of *Corylus* soil microorganisms change with the seasons.

## 2. Materials and Methods

### 2.1. Study Area and Soil Sampling

The study area was in the experimental station of Jiuxian Town, Yanqing District, Beijing, China, which has a continental monsoon climate with an annual average temperature of 8 °C and annual sunshine of 2800 h. In this area, the average temperature and precipitation in July is 22.01 °C and 110.45 mm, respectively, while that in January is −6.83 °C and 0.03 mm, respectively (Figure S1). In 2014, the experimental station prepared the land and introduced *C. heterophylla* (PZ), *C. kweichowensis* (CZ), *C. avellane* (OZ), and *C. heterophylla* × *C. avellane* (ZJ) with the same growth. The same management method was adopted for all four species. There were three randomly arranged plots of 10 m × 10 m for each species, and the row spacing of hazel trees was 2 m × 3 m. According to the World Reference Base for Soil Resources, the orchard soil type was mainly loam [23]. All four species of *Corylus* had 750 kg urea fertilizer ha-1 and 1000 kg manure compost ha-1 applied each year.

Soil samples were collected from the four hazelnut species after 5 years in April, July, and October of 2019 and in January 2020. For sampling, six trees were selected from each species, the soil at 20 cm distance from the tree, and 20 cm depth was collected and mixed in four directions. Finally, the soils of six trees in each plot were mixed as one sample. A total of 60 samples (5 treatments (CK (control), PZ, CZ, OZ, ZJ) × 4 seasons (spring, summer, autumn, winter) × 3 replicates) were obtained. After collection, soils were immediately put it into sterile plastic bags, placed into an incubator filled with dry ice, and transported to the laboratory. Soils were then divided into three parts, one part was stored at −80 °C, the second part was air-dried to analyze physicochemical properties, and the third part was used to measure soil water content and pH.

### 2.2. Soil Physicochemical Properties

Soil pH was measured by a pH meter (Mettler-Toledo, S40 SevenMulti™, Greifensee, Switzerland) with a 2.5:1 ratio of water to soil [24]. The soil water content (SWC) was determined according to the soil physical and chemical analysis [25]. Total organic carbon (TOC) content was determined by the K2CrO4 oxidation method, the total nitrogen (TN) content was measured by the Kjeldahl method, and the total phosphorus (TP) content was measured by the NaOH alkali fusion–atomic absorption method. Available phosphorus (AP) was determined by the Olsen method and available potassium (AK) was measured using a flame photometer after NH4OAc extraction [26].

### 2.3. DNA Extraction and PCR Quantification

Microbial DNA was extracted from soil samples using the E.Z.N.A.® Soil DNA Kit (Omega Bio-tek, Norcross, GA, USA) according to manufacturer’s protocols. The internal transcribed spacer (ITS) sequence was amplified with primers ITS1F (5′-CTTGGTCATTTAGAGGAAGTAA-3′) and ITS2R (5′-GCTGCGTTCTTCATCGATGC-3′), and the 16S rDNA gene sequence was amplified with primers 799 F (5′-AACMGGATTAGATACCCKG-3′) and 1193 R (5′-ACGTCATCCCCACCTTCC-3′) [27]. PCRs were performed in triplicate in 20-μL reactions containing 4 μL of 5× FastPfu Buffer, 2 μL of 2.5 mM dNTPs, 0.8 μL each primer (5 μM), 0.4 μL FastPfu Polymerase, and 10 ng template DNA. The amplification process consisted of an initial denaturation at 95 °C for 2 min, followed by 25 cycles at 95 °C for 30 s, 55 °C for 30 s, and 72 °C for 30 s, and a final extension at 72 °C for 5 min. Amplicons were extracted from 2% agarose gels and purified using the AxyPrep DNA Gel Extraction Kit (Axygen Biosciences, Union City, CA, USA) according to the manufacturer’s instructions and were quantified using QuantiFluor™-ST (Promega, USA). The NEXTflexTM Rapid DNA-Seq Kit (Bioo Scientific, USA) was used to build the database. The steps of building the database are divided into four steps: (1) linker linking; (2) using magnetic beads to screen and remove the linker self-connected fragments; (3) enriching the library template by PCR amplification; and (4) recovering PCR products by magnetic beads to obtain a final library. Sequencing was carried out by using Miseq PE300 platform of Illumina Company. The fastp software (https://github.com/OpenGene/fastp, version 0.20.0, accessed on 1 July 2021) was used for quality control of the original sequencing sequence, and the FLASH software (http://www.cbcb.umd.edu/software/flash, version 1.2.7, accessed on 15 March 2020) was used for splicing. According to the similarity of 97%, UPARSE (http://drive5.com/uparse/, version 7.1, accessed on 15 March 2020) was used to check chimera sequences. Use RDP classifier (http://rdp.cme.msu.edu/, version 2.2, accessed on 15 March 2020) to annotate each sequence for species classification. All sequence data were deposited in the NCBI Sequence Read Archive (SRA) database under accession number SRP313385 and BioProject ID PRJNA719642.

### 2.4. Ecological Niche Modeling

Maxent (https://biodiversityinformatics.amnh.org/open_source/maxent/, version 3.4.1, accessed on 24 March 2020) was used to predict the distribution area of *C. kweichowensis* from 2041 to 2060; climate data from 2041 to 2060 was from WorldClim (http://www.worldclim.com/, Version 1.4, accessed on 24 March 2020), and general circulation model simulations were obtained using the Community Climate System Model (CCSM) [28]. Distribution records for *C. kweichowensis* were sourced from the Chinese Virtual Herbarium (http://www.cvh.ac.cn/, accessed on 24 March 2020) and previously published papers [29,30,31]. For *C. kweichowensis*, seven uncorrelated (|r| ≤ 0.8) and biologically significant bioclimatic variables were selected as predictors: (1) annual mean temperature; (2) mean diurnal range; (3) isothermality; (4) temperature seasonality; (5) mean temperature of wettest quarter; (6) annual precipitation; and (7) precipitation seasonality. Twenty-five percent of the distribution data of *C. kweichowensis* was randomly selected as the test set, and the remaining data were the training set. Bootstrapping was repeated 10 times.

### 2.5. Statistical Analysis

Statistical analysis of operational taxonomic unit (OTU) richness, shannon’s, evenness, and good’s coverage index was performed with Mothur (version 1.46.1, https://github.com/mothur/mothur/releases/tag/v1.46.1, accessed on 17 May 2020). One-way analysis of variance (ANOVA) followed by Duncan’s multiple range test (DMRT) was carried out by SPSS version 26.0 (SPSS Inc., Chicago, IL, USA) to assess the significance of the effects of seasons on soil properties and diversity. Redundancy analysis (RDA) was conducted using Canoco (version 4.5 for Windows; Ithaca, NY, USA) with forward selection based on Monte Carlo permutations (permu = 999) to reflect the relationship among samples, soil physicochemical properties, and bacterial community; the variance inflation factor (VIF) values of C/N were higher than 10 and thus were eliminated. Functions of fungal communities were classified and analyzed by FUNGuild (http://www.stbates.org/guilds/app.php, accessed on 17 May 2020), with the fungi divided into pathotrophs, symbiotrophs, and saprotrophs [32]. The fungi in the analysis were the species that belong to a single guild [33]. In this study, Animal Pathogen, Plant Pathogen, Undefined Saprotroph, Dung Saprotroph, Wood Saprotroph, and Ectomycorrhizal that contained more than 1% of species were predominantly selected. PICRUSt2 (https://github.com/picrust/picrust2, accessed on 17 May 2020) was used to predict the functional potential of bacteria [34]. Co-occurrence network analyses of the bacterial communities were conducted using the Python package ‘networkx’ [35]. According to previous studies, association analysis and co-occurrence network analysis are not suitable for rare species; therefore, only the top 50 genera were selected for network analysis [36].

## 3. Results

### 3.1. Soil Physicochemical Properties

**Figure 1:**
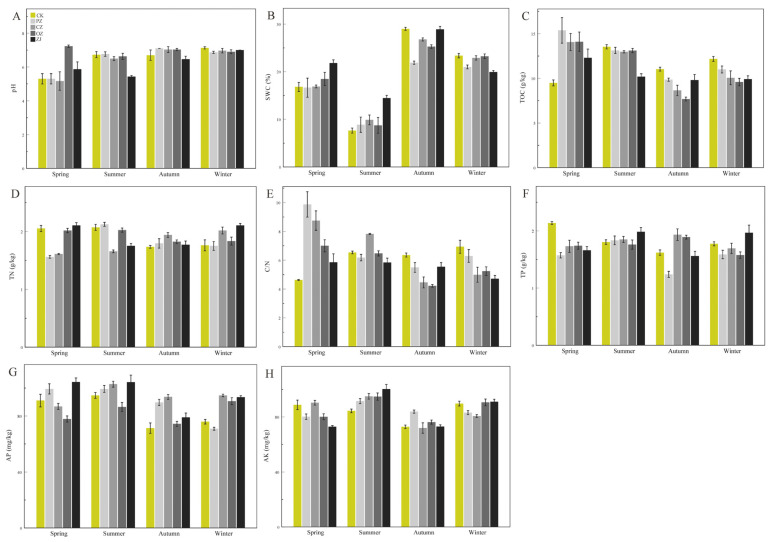
Soil physicochemical properties of four species of the genus *Corylus* across different seasons. (**A**) pH; (**B**) SWC; (**C**) TOC content; (**D**) TN content; (**E**) C/N content; (**F**) TP content; (**G**) AP content; (**H**) AK content. SWC: soil water content; TOC: total organic carbon; TN: total nitrogen; C/N: ratio of C and N; TP: total phosphorus; AP: available phosphorus; AK: available potassium; CK: control; PZ: *C*. *heterophylla*; CZ: *C*. *kweichowensis*; OZ: *C*. *avellane*; ZJ: *C*. *heterophylla* × *C*. *avellane*.

The soil physicochemical properties of the four species of the genus *Corylus* in the four seasons are depicted in Figure 1. Soil pH ranged from 5.17 (CZspr) to 7.23 (OZspr) (Figure 1A, Table S1), and the pH for *C*. *heterophylla* (PZ) in the spring was significantly different from that of the other three seasons (Table S2). There were marked differences in pH between seasons for *C*. *kweichowensis* (CZ) except for autumn and winter. The pH for *C*. *avellane* (OZ) was significantly different in the summer compared with the other three seasons, and there were significant differences among the four seasons for *C*. *heterophylla* × *C*. *avellane* (ZJ) (*p* < 0.05, Table S2). There was no significant difference in soil pH between the four species of *Corylus* in the winter, and all species except OZ (pH 7.23) had a lower pH in the spring (Table S2). SWC for the four species of *Corylus* showed significant differences in each season (*p* < 0.05, Table S2). SWC for all four species was lowest in the summer and showed a trend of decreasing, then increasing, and then decreasing again (Figure 1B). TOC displayed a similar trend of decreasing first and then increasing, and it reached the minimum value in autumn (Figure 1C). TN values of CZ and OZ were not significantly different between spring and summer, while PZ and ZJ exhibited the opposite (*p* < 0.05, Table S2). There was also no significant difference between autumn and winter for CZ, OZ, and PZ (Figure 1D, Table S2). Except for ZJ, the value for the C/N ratio decreased between spring and autumn, and then increased in winter (Figure 1E). Soil TP values of CZ and OZ increased initially between spring and autumn and then decreased in winter, while the values of PZ and ZJ tended to increase between spring and summer, then decreased in autumn, and increased again in winter (Figure 1F). The AP values of CZ and OZ increased between spring and summer, decreased in autumn, and then increased again in winter, while the AP values of PZ showed a continual decrease through the seasons from spring to winter, and those of ZJ decreased from summer to autumn, and then increased in winter (Figure 1G). The AK values of the four species increased initially between spring and summer, then decreased in autumn, before increasing again in winter (Figure 1H). There were significant differences in AK values between CZ and OZ in the four seasons (Table S2).

### 3.2. Soil Microbial Diversity

**Figure 2:**
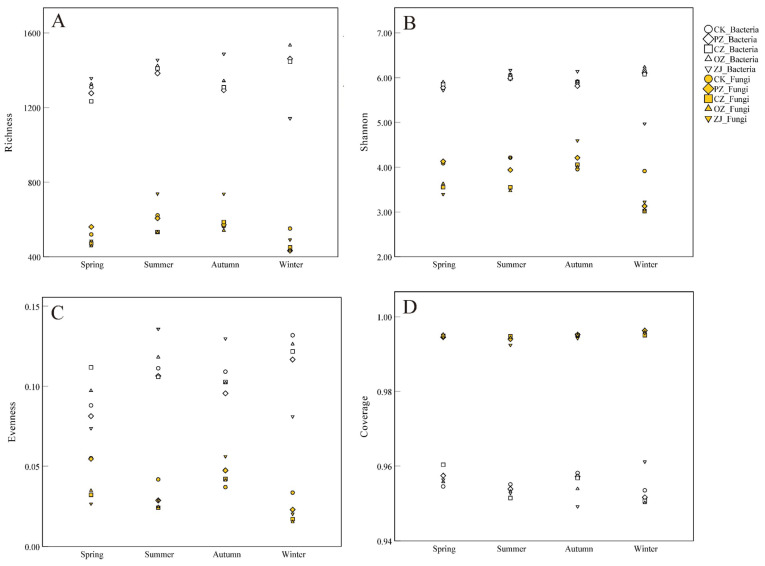
Diversity indices of soil microbial communities. (**A**) OTU richness index; (**B**) Shannon index; (**C**) Community evenness; (**D**) Community coverage. CK: control; PZ: *C*. *heterophylla*; CZ: *C*. *kweichowensis*; OZ: *C*. *avellane*; ZJ: *C*. *heterophylla* × *C*. *avellane*.

As shown in Figure 2, the fungal OTU richness index ranged from 432.7 (PZwin) to 738.3 (ZJsum), while the bacterial OTU richness index ranged from 1141.3 (ZJwin) to 1533.3 (OZwin) (Table S3). The OTU richness index of fungi in PZ soil in the winter was significantly different from that of the other three seasons (*p* < 0.05), and the OTU richness index of fungi in CZ soil in winter was significantly different from that in autumn (*p* < 0.05). There were significant differences between ZJspr and ZJwin, as well as ZJsum and ZJaut (*p* < 0.05). Except for the significant differences between ZJsum, ZJaut, and the other two seasons, there were no significant differences in the fungal OTU richness index among different species of *Corylus* in the same season. In bacteria, except ZJwin, there were no significant differences in OTU richness index among different species of *Corylus* in the same season (Table S3). The seasonal variation trend in the OTU richness index of fungi was an initial increase between spring and autumn, followed by a decrease in winter, while for bacteria, the trend in PZ, CZ and OZ was an initial increase between spring and summer, then a decrease in autumn, and an increase again in winter. In contrast, ZJ showed an increase in bacterial OTU richness index between spring and autumn before decreasing in winter. Significant differences and trends in the Shannon index of bacteria among samples were similar to those of the OTU richness index. Bacterial evenness indices of CK, PZ, CZ, and OZ showed no significant differences in each season, but there was an obvious difference between summer and autumn for ZJ. The bacterial evenness index trend of CZ was an initial decrease between spring and autumn followed by an increase in winter. In contrast, ZJ displayed an initial increase between spring and summer and then decreased, and the other three samples (CK, PZ, and OZ) increased between spring and summer, decreased in autumn, and then increased again in winter. The whole seasonal variation trend of evenness index of fungi was opposite to that of bacteria. All samples had good coverage, and the average value was above 0.95 (Fungi: 0.99; Bacteria: 0.95). The richness index, Shannon index, and evenness index values of bacteria were higher than those of fungi.

### 3.3. Soil Microbial Community Structure and Function

**Figure 3:**
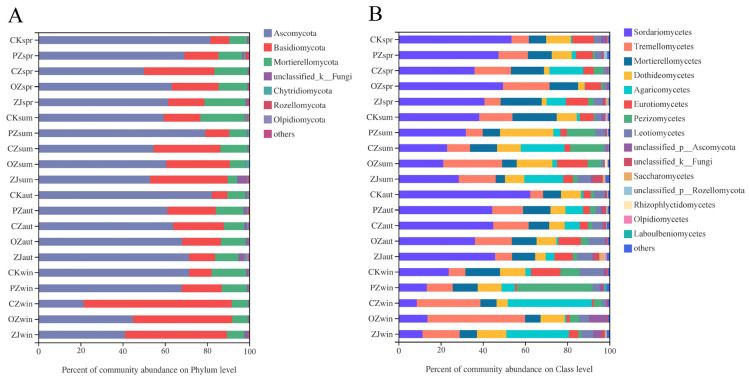
Relative abundances of soil fungal community structure at phylum (**A**) and class (**B**) levels. CK: control; PZ: *C*. *heterophylla*; CZ: *C*. *kweichowensis*; OZ: *C*. *avellane*; ZJ: *C*. *heterophylla* × *C*. *avellane*; spr: spring; sum: summer; aut: autumn; win: winter.

The predominant fungi phyla in all samples were Ascomycota (66.21%), Basidiomycota (22.82%), and Mortierellomycota (9.32%) (Figure 3A). Seasonal variation of Ascomycota content occurred in OZ and ZJ soils with an initial decrease between spring and summer, followed by an increase in autumn, and then a decrease in winter, while PZ showed the opposite trend, and CZ increased between spring and autumn and then markedly decreased in winter. The seasonal variation trend of Basidiomycota and Mortierellomycota contents in PZ, OZ, and ZJ soils were consistent with the variation trend of Ascomycota content in OZ soil. At the class level of fungi, the main classes in all samples were *Sordariomycetes* (34.74%), *Tremellomycetes* (15.62%), and *Mortierellomycetes* (11.70%) (Figure 3B). Seasonal variation of *Sordariomycetes* in all samples comprised an initial decrease from spring to summer, then an increase in autumn, followed by a decrease in winter. Seasonal variation of *Tremellomycetes* content in PZ soil was consistent with that of *Sordariomycetes* content, but contrary to the seasonal variation of *Tremellomycetes* content observed for OZ and ZJ. *Tremellomycetes* content in CZ soil decreased between spring and summer and then increased from autumn onwards. Seasonal variation of *Mortierellomycetes* content in each sample was consistent with that of the phylum Mortierellomycota.

**Figure 4:**
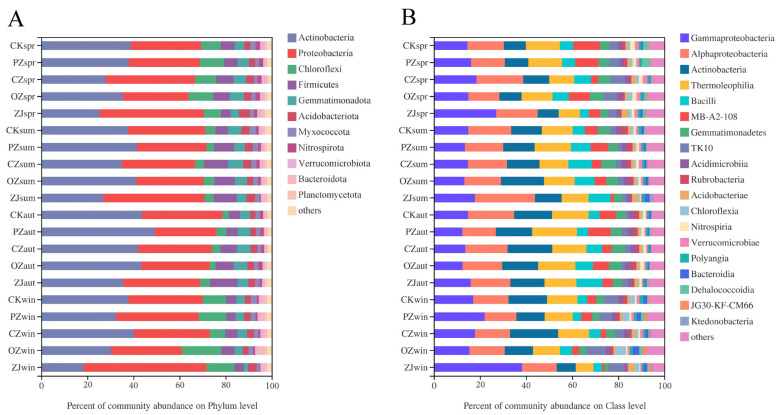
Relative abundances of soil bacterial community structure at phylum (**A**) and class (**B**) levels. CK: control; PZ: *C*. *heterophylla*; CZ: *C*. *kweichowensis*; OZ: *C*. *avellane*; ZJ: *C*. *heterophylla* × *C*. *avellane*; spr: spring; sum: summer; aut: autumn; win: winter.

At the bacterial phylum level, the dominant taxa in all samples were Actinobacteria (36.08%) and Proteobacteria (34.22%) (Figure 4A). The seasonal variation trend of Actinobacteria content of all four species of *Corylus* was an initial increase between spring and autumn, followed by a decrease in winter. The seasonal variation trend of Proteobacteria content in PZ, CZ, and ZJ soils was an initial decrease between spring and autumn, followed by an increase in winter, while OZ showed a continuous increase throughout the seasons from spring to winter. At the bacterial class level, the dominant taxa in all samples were *Gammaproteobacteria* (17.39%), *Alphaproteobacteria* (16.95%), *Actinobacteria* (13.40%), and *Thermoleophilia* (13.28%) (Figure 4B). *Gammaproteobacteria* content in the soil of all four species of *Corylus* initially decreased between spring and autumn, and then increased in winter. Seasonal variation trends of the content of the class *Alphaproteobacteria* in PZ, OZ, and ZJ samples were consistent with those of *Gammaproteobacteria*. The seasonal variation trend of *Actinobacteria* content in CZ soil is a continuous increase, reaching the maximum in winter, while PZ, OZ and ZJ all increased initially and then decreased, reaching the maximum in summer (OZ) and autumn (PZ, ZJ), respectively.

There were significant differences (*p* < 0.05) in the class level (the first 15 classes) of soil fungi of all four species of the genus *Corylus* in the spring, summer, and winter, while for bacteria, significant differences were observed at the class level for all four species of *Corylus* in all seasons except spring (Figures S2 and S3). In spring, the class of soil fungi with significantly different abundance between the four species of *Corylus* was *Saccharomycetes*, while in summer, the significantly different fungal classes were *Sordariomycetes*, *Agaricomycetes*, *unclassified_p__Ascomycota*, *Saccharomycetes*, and *Zoopagomycetes*, and in winter, they were *Agaricomycetes*, *Eurotiomycetes*, *Taphrinomycetes*, and *Microbotryomycetes*. Among the bacteria, *MB-A2-108*, *Rubobacter*, Acidobacteriae, *Verrucomicrobiae*, and *Holophagae* were the classes with significantly different abundances in soil between the species of *Corylus* in summer. In autumn, the significantly different classes were *Bacilli* and *Rubrobacteria*, and in winter, *Gammaproteobacteria* was the only class displaying a significant difference in abundance among soil samples of the species of *Corylus*.

**Figure 5:**
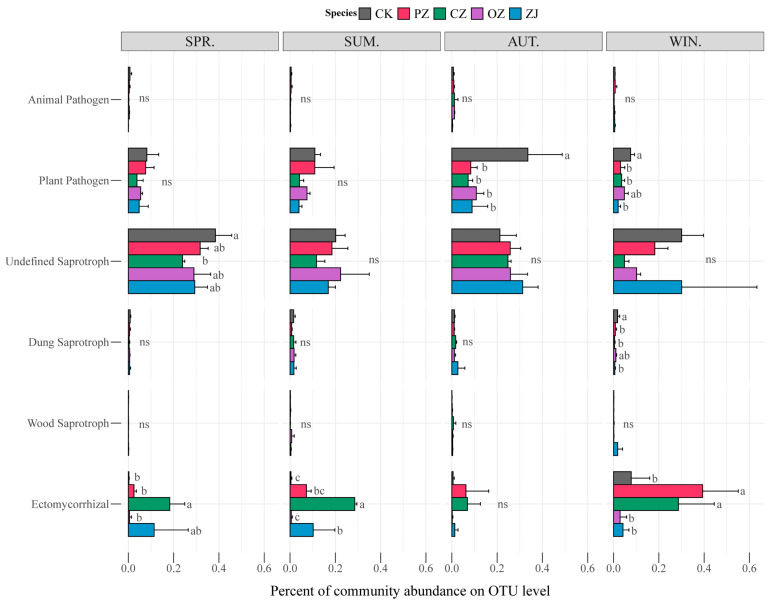
Functional features of fungal communities in four species of the genus *Corylus* in different seasons. CK: control; PZ: *C*. *heterophylla*; CZ: *C*. *kweichowensis*; OZ: *C*. *avellane*; ZJ: *C*. *heterophylla* × *C*. *avellane*; spr: spring; sum: summer; aut: autumn; win: winter. Different letters (a,b) indicate the significance level at *p* < 0.05, ns indicate no significance (*p* > 0.05).

The seasonal changes of soil fungal function for each species of the genus *Corylus* sampled in this study are shown in Figure 5. The functions of animal pathogen and plant pathogen belong to pathotroph; undefined saprotroph, dung saprotroph, and wood saprotroph belong to saprotroph; and ectomycorrhizal belongs to symbiotroph. The pathotroph guild was dominated by the function of plant pathogen, while saprotroph was dominated by undefined saprotroph. Statistical analysis of the guilds revealed that there were no significant differences among the four species of *Corylus* in each season except for the ectomycorrhizal guild, which showed an obvious change in abundance with the seasons. In spring and summer, the ectomycorrhizal abundance of CZ was significantly higher than that of other species of the genus *Corylus*, and in winter, the ectomycorrhizal abundance of PZ and CZ was significantly higher than that of OZ and ZJ. According to the prediction results of bacterial function by PICRUSt2, there were no significant differences among the four species of the genus *Corylus* examined in this study (Figure S4).

### 3.4. Relationships of Microbial Communities and Soil Properties

**Figure 6:**
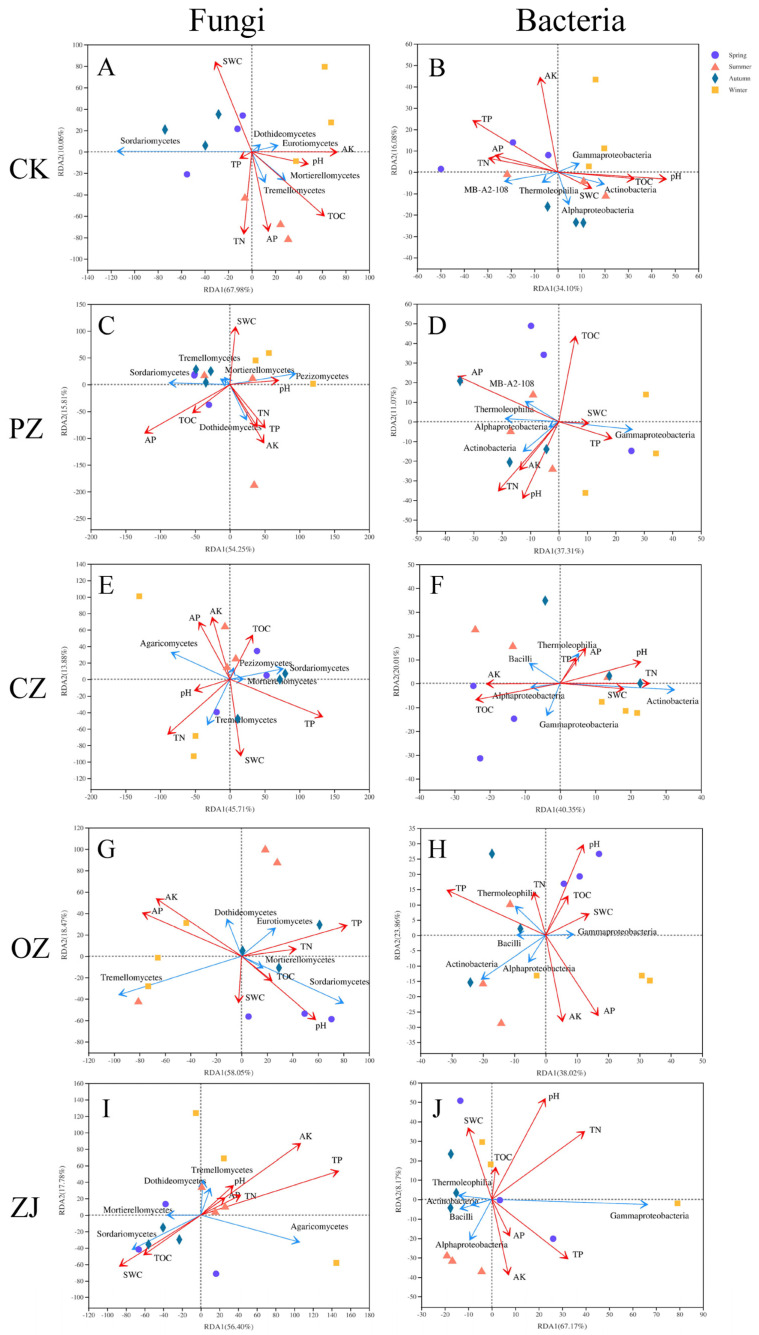
Redundancy analysis (RDA) of the top five fungal and bacterial classes with soil proper ties. RDA of top five fungal classes with soil properties of CK (**A**), PZ (**B**), CZ (**C**), OZ (**D**), and ZJ (**E**) samples. RDA of top five bacterial classes and soil properties of CK (**F**), PZ (**G**), CZ (**H**), OZ (**I**), and ZJ (**J**) samples. CK: control; PZ: *C*. *heterophylla*; CZ: *C*. *kweichowensis*; OZ: *C*. *avellane*; ZJ: *C*. *heterophylla* × *C*. *avellane*.

AP was the major driving factor of soil fungal community in PZ (*p* < 0.05), while pH, TOC, and TN were the predominant driving factors of bacterial community composition in CZ (*p* < 0.05). TP and AP had significant effects on both fungal and bacterial communities in OZ (*p* < 0.05). In addition, pH and AK were also the main environmental drivers of fungal communities in OZ (*p* < 0.05). TP and AK were the main environmental drivers of fungal community composition in ZJ (*p* < 0.05), while pH and TN had significant effects on bacterial community composition in ZJ (*p* < 0.05) (Table S4). In soil microorganisms, there were also strong correlations among environmental factors. For example, in soil fungi of PZ and ZJ, there was a strong correlation between AP and TN, and in soil bacteria of OZ and ZJ, there was a strong correlation between pH, TOC, and SWC (Figure 6). Correlation analysis between environmental factors and the first 20 classes of fungi and bacteria (Figure 6 and Figure S7) indicated that AK was the predominant environmental factor affecting fungal community composition and TP was the main one affecting bacterial community composition.

### 3.5. Co-Occurrence Network Characteristics

**Figure 7:**
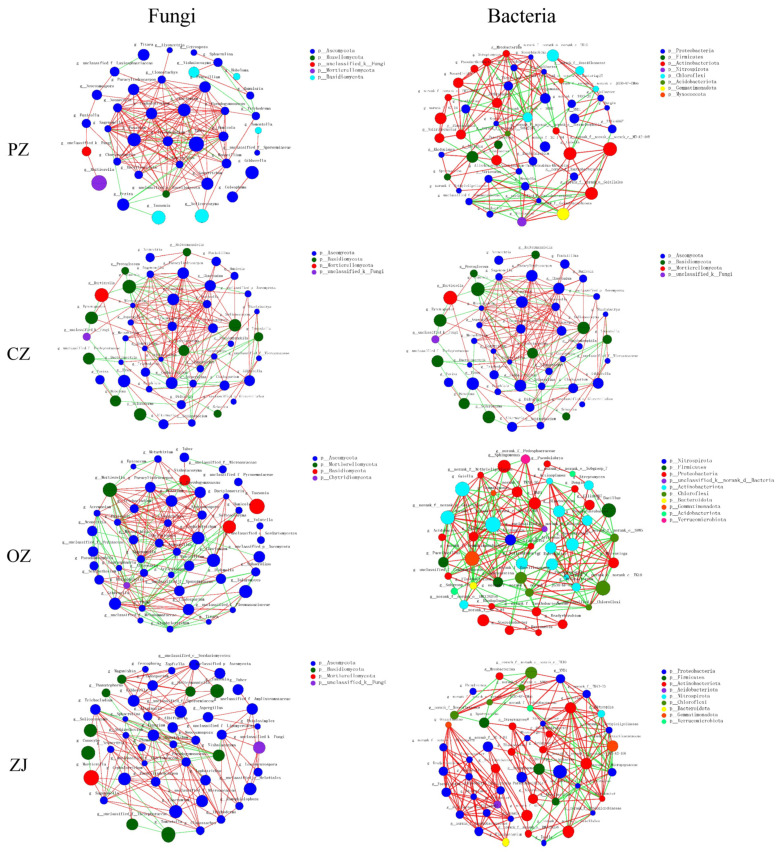
Co-occurrence network of fungal and bacterial communities across four species of the genus *Corylus* based on correlation analysis. Nodes in the networks are colored by phylum. Connections represent strong (Spearman’s ρ > 0.6) and significant (*p* < 0.05) correlations. The edge color represents positive (red) and negative (green) correlations. The size of each node is proportional to the relative abundance of a specific genus. The thickness of each edge is proportional to the ρ. PZ: *C*. *heterophylla*; CZ: *C*. *kweichowensis*; OZ: *C*. *avellane*; ZJ: *C*. *heterophylla* × *C*. *avellane*.

Table 1 and Table 2 shows the co-occurrence network of soil microorganisms in four species of the genus *Corylus* and in four seasons based on significant correlations. There were significant differences in microbial networks among the four species of *Corylus*. There was no obvious relationship between the abundance of microbial genera and their importance in the network (Figure 7). At the fungal level, the OZ network has the most edges and ZJ network has the least. However, at the bacterial level, the ZJ network has the most edges while the PZ network had the least. The law of average connectivity was consistent with that of edges. The highest clustering coefficient value for fungi among the four species of *Corylus* was in PZ, but this species displayed the lowest clustering coefficient value for bacteria. The positive interaction of PZ and ZJ in fungi was 5.58 and 5.61 times higher, respectively, than that in negative interaction, while it was 2.06 and 4.31 times higher, respectively, in bacteria.

Seasonal variation occurred in the number of edges in fungi, with an increase from spring to summer, and then decreasing from summer onwards; the highest value was observed in summer and the lowest value in winter. For bacteria, the number of edges increased from spring to summer, then decreased in autumn, and increased again in winter. Like fungi, the peak of the number of edges for bacteria was also reached in the summer, but the lowest value was observed in autumn. The trend of average connectivity for bacteria was consistent with that of the number of edges. The maximum value for fungal clustering coefficient was reached in autumn and the minimum in winter, but the clustering coefficient values for bacteria showed the opposite trend (Table 1 and Figure S4). In fungi, the positive interactions in spring and winter were 1.64 and 2.62 times greater than that of negative interactions, respectively, and in bacteria, they were 2.04 and 6.32 times greater, respectively (Table 1 and Figure S5).

### 3.6. Future Distribution of C. kweichowensis

According to the sequencing results, Tuber was the main ectomycorrhizal fungal genus that was symbiotic with *C. heterophylla* and *C. kweichowensis*. The characteristics of the genus *Tuber* indicates that this fungus mainly inhabits southern China, and *C. kweichowensis*, which is also distributed in southern China, can therefore be used as an indicator species to predict the potential distribution area of Tuber. Furthermore, to clarify the suitability of *C. kweichowensis* for culture in China, only the distribution records of *C. kweichowensis* in China were considered for niche modeling. After modeling, the average area under the curve was 0.991 (Figure S8), indicating that the model had a high simulation value. The model, based on forecasted climatic conditions in 2041–2060 (Figure S9), indicated that *C. kweichowensis* had good suitability in central Jiangsu, southern Shaanxi, southeastern Gansu, northwestern Hubei, eastern Sichuan, and central Guizhou, areas that were mainly concentrated in the mountains around the Sichuan Basin in China.

## 4. Discussion

Water is one of the key environmental parameters and is an important variable affecting microbial community structure and carbon and nitrogen transformation [37,38,39,40,41]. However, in the current study, SWC did not significantly affect the changes in microbial community structures (Figure 6, Figures S6 and S7). In the current study, AK is hypothesized to be the main environmental factor driving the change in fungal community, while TP is the one driving bacterial community changes. In general, the ability of plant roots to absorb water and nutrients will be limited under acidic conditions, thus inhibiting the growth and development of plants [42]. The variation trend of pH with SWC, TOC, and C/N in the present study demonstrated that acidic conditions were not conducive to the absorption of water and nutrients for the four species of the genus *Corylus*. Among them, the autumn value of SWC was higher, which may be due to precipitation a few days before sampling, leaf litter and low temperature blocking the evaporation of water. TOC and TN also had important effects on soil microbial community composition [43,44].

Seasonal changes can affect the diversity of microorganisms [45,46,47,48], and in general, the richness of bacteria in the same habitat is higher than that of fungi. The diversity of bacteria in this study was significantly higher than that of fungi (Figure 2), congruent with previous studies [20,49]. There were significant differences in fungal diversity among seasons, but there was no significant difference in bacterial diversity among seasons (Table S2). Bacteria have a wider range of life and often form biofilms in the soil [50]. Therefore, fungi are more susceptible to precipitation and temperature changes caused by seasonal changes than bacterial communities; hence fungi and bacteria have different adaptability to environmental changes [20,38]. The change trend of fungal Shannon diversity in the current study was similar to that of He et al. [20], and the change trend of bacterial diversity was also similar to previous studies [49,51]. The increase in soil moisture caused by summer precipitation may be one reason for the increase in microbial richness [37,40]. The nutrient supply in autumn was related to the decrease in total bacterial community diversity during these periods, and this may be due to dry conditions and limited nutrient conditions that were previously observed to result in decreased diversity in October [52,53]. A previous study showed that seasonal changes in photosynthesis had a greater impact on soil respiration compared with seasonal changes in soil temperature, and that a decrease in soil respiration and soil temperature leads to a decrease in microbial diversity [54]. However, in the current study, bacterial diversity did not decrease in the winter. This may be due to the input of litter, which increases organic matter in the soil [55,56]. In addition, after entering autumn, precipitation markedly decreased in the present study, and because fungi are more susceptible to drought stress than bacteria, the diversity of fungi will therefore decrease [20,50]. Another possible reason for the increased bacterial diversity in the current study is that the soil in the area sampled was not very sensitive to the above factors, and another potential explanation is that plants have less demand for soil nutrients in winter, therefore bacterial diversity and abundance will increase due to the availability of nutrients for the bacteria.

The community composition of fungi and bacteria showed obvious seasonal changes (Figure 3 and Figure 4). Season was a key driving force of soil microorganisms, and this was in agreement with previous reports [57]. According to reported studies, some members of Agaricomycetes are related to ectomycorrhiza. Ectomycorrhiza can promote the growth of trees [58]. *C*. *heterophylla* (PZ), *C*. *kweichowensis* (CZ), and *C*. *heterophylla* × *C*. *avellane* (ZJ) all had a high proportion of *Agaricomycetes* in each season. This was consistent with the functional abundance of symbiotic bacteria predicted by FUNGuild in Figure 5. Sequencing results indicated that the main ectomycorrhizal genera of the class *Agaricomycetes* were *Hymenogaster*, *Scleroderma*, *Hebeloma*, *Tomentella*, and *Tuber*. PZ and CZ had more ectomycorrhizal symbionts than *C. avellane* (OZ) had. Among them, *Tuber* is a rare edible fungus with important nutritional and economic value [59], including several species of truffles. Most truffles coexist with trees or shrubs. Previous studies on the mycorrhizal effect of truffle on *C. avellane* seedlings showed that *C. avellane* can coexist with truffle and that truffle can improve the rooting rate and root length of the hazel cuttings [60,61,62]. Truffles mainly grow in southwest China; therefore, the prediction of suitable areas for culture of *C. kweichowensis* based on climate forecasting data from 2041 to 2060 can not only identify potential suitable areas for truffles but can also provide a theoretical basis for the establishment of *C. kweichowensis*-truffle cultivation gardens.

The content of Actinobacteria in the soil of all hazel species was the highest in autumn and showed obvious seasonal changes, which was congruent with other research results [7,63]. Actinobacteria could decrease in response to plant root exudates, and this explains the low content of actinomycetes in most species of the genus *Corylus* in the spring and summer [64]. However, the abundance of Actinobacteria in OZ increased in the summer, which may be due to reduced secretion of root exudates in the summer. Root exudates play an important role in soil physical and chemical properties and the construction of soil microbial communities [65]. *C. avellane*, which is a subtropical tree species and likes a humid climate, was introduced from Europe to the experimental site. Climatic conditions of a warm and humid winter and a dry summer are most beneficial to the growth and fruiting of this species [66]. Therefore, it may be that *C. avellane* was not suited to the local climate of the experimental site and, consequently, cannot form a symbiotic system with more ectomycorrhiza, which in turn may also make *C. avellane* more unsuitable for the local climate. A possible solution to consider may be inoculating ectomycorrhiza from *C. heterophylla* and *C. kweichowensis* into *C. avellane*. The unsuitability of *C. avellane* to the local climate was also demonstrated by the co-occurrence network analysis. In general, more positive linear correlations indicate that a network structure is more stable [67,68]. Therefore, compared with *C. heterophylla* and Ping’ou (*C. heterophylla* × *C. avellane*), the network structure of *C. avellane* was more susceptible to the environment. Complex networks with higher connectivity have been proven to be more stable and less susceptible to environmental disturbances than simple networks with lower connectivity [69,70]. With the change of seasons, bacteria had higher network complexity, connectivity, and positive linear correlation than fungi (Figure S5). Therefore, the bacterial community was relatively more stable than the fungal community, and this was consistent with the regularity reflected previously by diversity.

## 5. Conclusions

This study employed sequencing and analysis of microbial genes to clarify the seasonal variation trends of the soil microbial community of four species of the genus *Corylus* and elucidate the main environmental factors driving microbial community composition. Fungi of phyla in different seasons were mainly Ascomycota, Basidiomycota, and Mortierellomycota, while bacteria were mainly Actinobacteria and Proteobacteria. AK may drive changes in fungal community, while TP may be responsible for bacterial community changes. The variation in rainfall induced by the seasons resulted in bacteria in soil having stronger adaptability than fungi, which was manifested in increased diversity, a complex co-occurrence network, enhanced connectivity, and greater positive linear correlation. There were abundant ectomycorrhizal fungi, especially *Tuber*, in soil microorganisms of *C. heterophylla* and *C. kweichowensis*, but few in *C. avellane*. This may be attributed to the fact that *C. avellane* in Europe was not suitable for the climate of the experimental site. There has been a focus on mycorrhiza because of its positive influence on plants. Consequently, investigating *Tuber* resources according to the predicted distribution area of *C. kweichowensis* and inoculating *Tuber* into hazelnut cultivation gardens to generate a symbiotic relationship may provide economic benefits.

## Figures and Tables

**Table 1:** Microbial network properties at four *Corylus* species.

	Network Metrics	CK	PZ	CZ	OZ	ZJ
Fungi	Number of nodes	46	48	47	48	46
	Number of edges	146	158	170	223	152
	Average connectivity	6.35	6.58	7.23	9.29	6.61
	Clustering coefficient	0.4	0.45	0.46	0.54	0.52
	Positive interaction	95	134	124	146	129
	Negative interaction	51	24	46	77	23
Bacteria	Number of nodes	49	48	49	48	48
	Number of edges	207	150	182	222	223
	Average connectivity	8.45	6.25	7.43	9.25	9.29
	Clustering coefficient	0.46	0.48	0.5	0.52	0.62
	Positive interaction	105	101	96	118	181
	Negative interaction	102	49	86	104	42

**Table 2:** Microbial network properties at four seasons.

	Network Metrics	Spring	Summer	Autumn	Winter
Fungi	Number of nodes	48	49	47	46
	Number of edges	127	188	116	94
	Average connectivity	5.29	7.67	4.94	4.09
	Clustering coefficient	0.36	0.4	0.45	0.33
	Positive interaction	79	98	57	68
	Negative interaction	48	90	59	26
Bacteria	Number of nodes	49	48	45	50
	Number of edges	173	235	126	183
	Average connectivity	7.06	9.79	5.6	7.32
	Clustering coefficient	0.48	0.52	0.37	0.53
	Positive interaction	116	126	76	158
	Negative interaction	57	109	50	25

## Data Availability

All sequence data were deposited in the National Center for Biotechnology Information (NCBI) Sequence Read Archive (SRA) database under accession number SRP313385 and BioProject ID PRJNA719642.

## Supplementary Materials

The following are available online at https://www.mdpi.com/article/10.3390/microorganisms9112228/s1, Figure S1: Precipitation and temperature of Yanqing in 2019. Figure S2: Significant difference between fungal groups. Figure S3: Significant difference between bacterial groups. Figure S4: The bacterial community functional features in four *Corylus* species in different seasons. CK: control; PZ: *C*. *heterophylla*; CZ: *C*. *kweichowensis*; OZ: *C*. *avellane*; ZJ: *C*. *heterophylla* × *C*. *avellane*; spr: spring; sum: summer; aut: autumn; win: winter. Figure S5: Co-occurring network of fungal and bacterial communities in different seasons. CK: control; PZ: *C*. *heterophylla*; CZ: *C*. *kweichowensis*; OZ: *C*. *avellane*; ZJ: *C*. *heterophylla* × *C*. *avellane*. Figure S6: Heatmap of correlation analysis of fungal environmental factors. Figure S7: Heatmap of correlation analysis of bacterial environmental factors. Figure S8: Receiver operator characteristic (ROC) curves of Maxent model. Figure S9: Modelled climatically suitable areas for the *C. kweichowensis* at 2041–2060. Table S1: Soil Properties of four *Corylus* species. Table S2: Significant differences in soil properties and microbial diversity of four *Corylus* species. Table S3: Microbial diversity of four *Corylus* species. Table S4: Redundancy analysis (RDA) of fungi and bacteria with soil properties of four *Corylus* species.
