# Supplementary material for: Seasonal Changes in Soil Microbial Community and Co-Occurrence Network of Species of the Genus Corylus
Source: Microorganisms. 2021 Oct 26;9(11):2228. doi: 10.3390/microorganisms9112228 (PMC8625130; doi:10.3390/microorganisms9112228)
Supplement: Supplementary file 1 [file microorganisms-09-02228-s001.zip › microorganisms-1380492-supplementary - proofreading-done.pdf]

microorganisms

MDPI

Supplementary Material

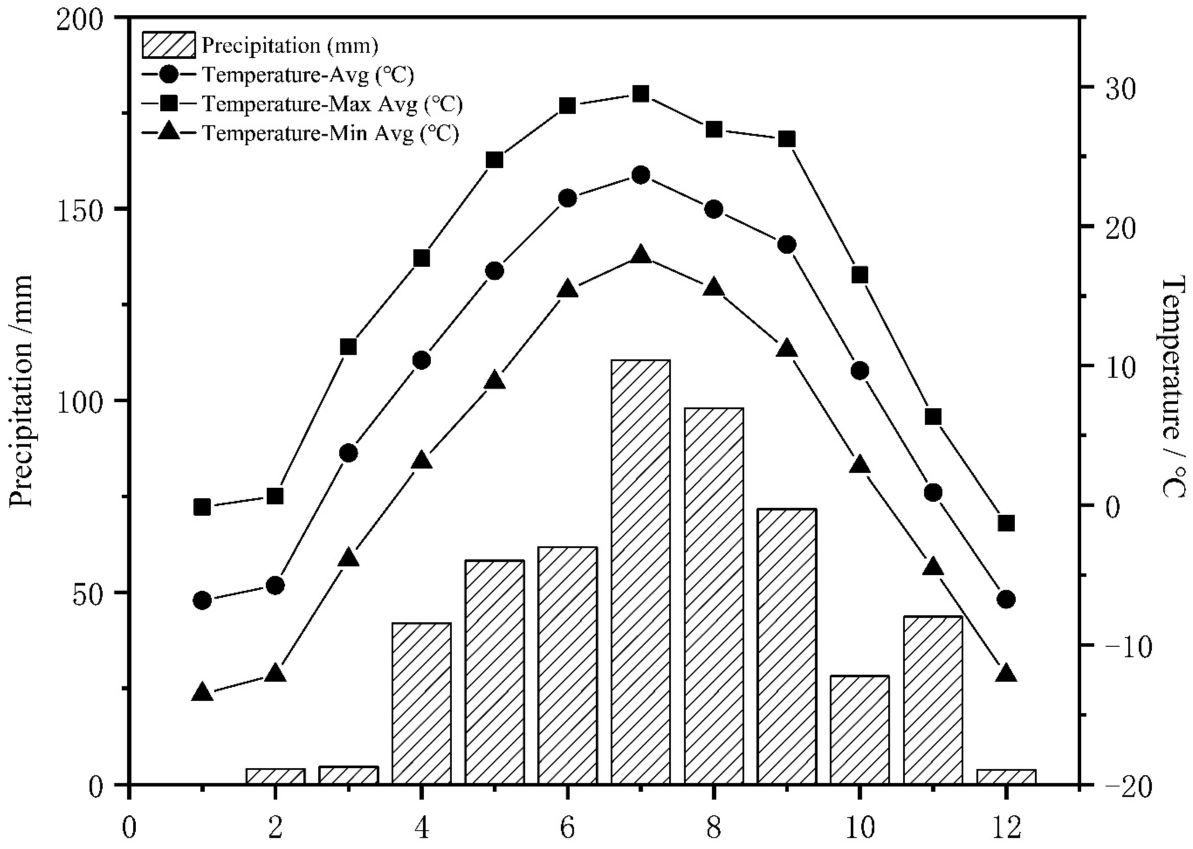

Figure S1. Precipitation and temperature of Yanqing in 2019.

Fungi

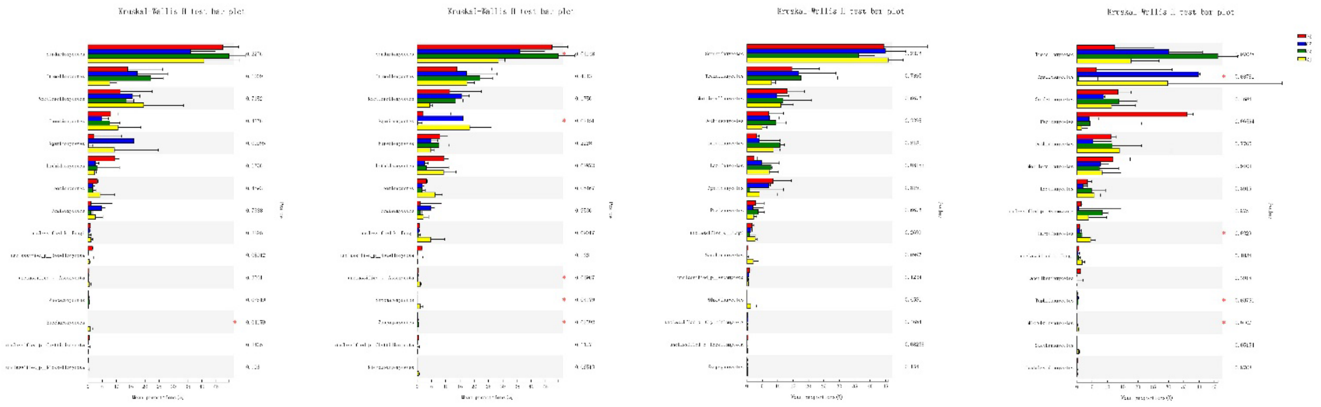

Spring Summer Autumn Winter

Figure S2. Significant difference between fungal groups.

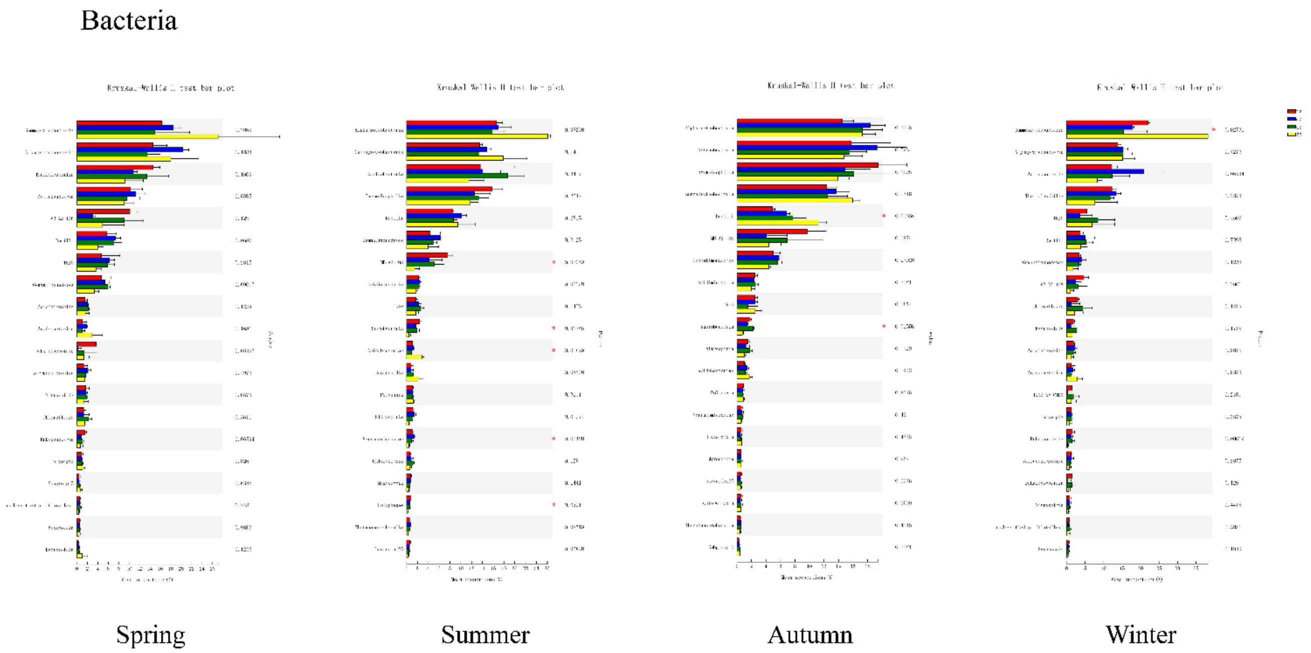

Figure S3. Significant difference between bacterial groups.

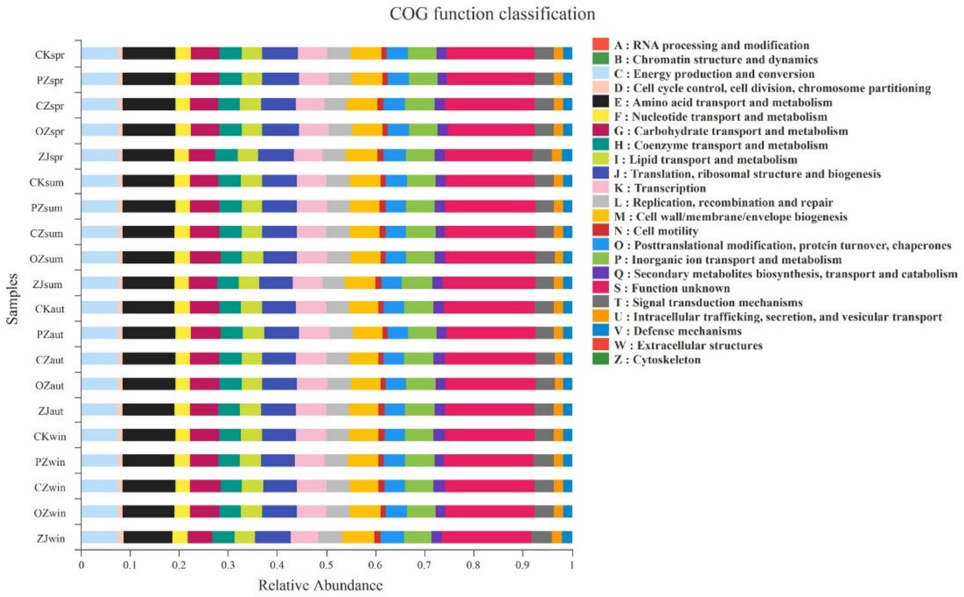

Figure S4. The bacterial community functional features in four Corylus species in different seasons. CK: control; PZ: C. heterophylla; CZ: C. kweichowensis; OZ: C. avellane; ZJ: C. heterophylla × C. avellane; spr: spring; sum: summer; aut: autumn; win: winter.

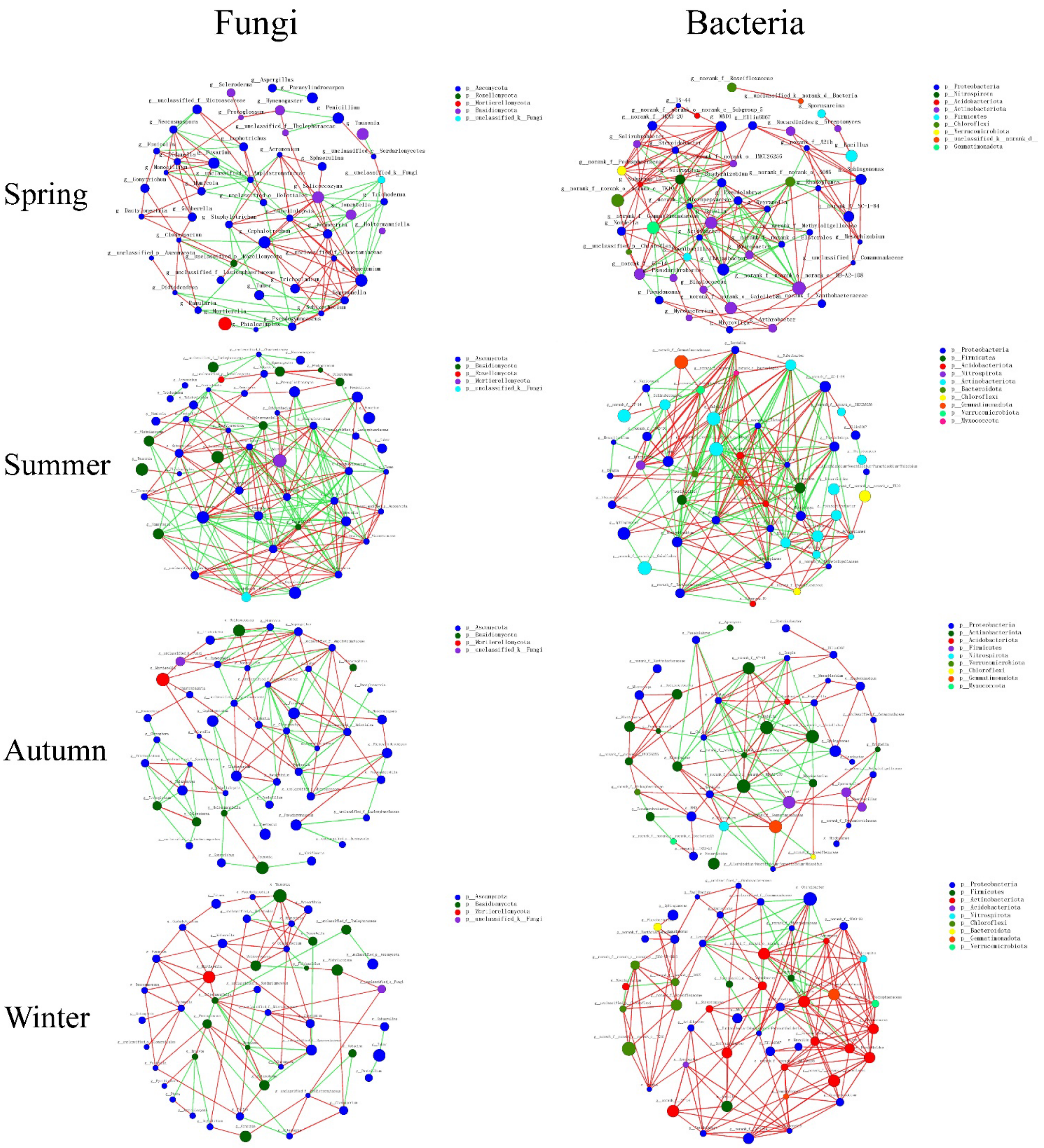

Figure S5. Co-occurring network of fungal and bacterial communities in different seasons. CK: control; PZ: C. heterophylla; CZ: C. kweichowensis; OZ: C. avellane; ZJ: C. heterophylla × C. avellane.

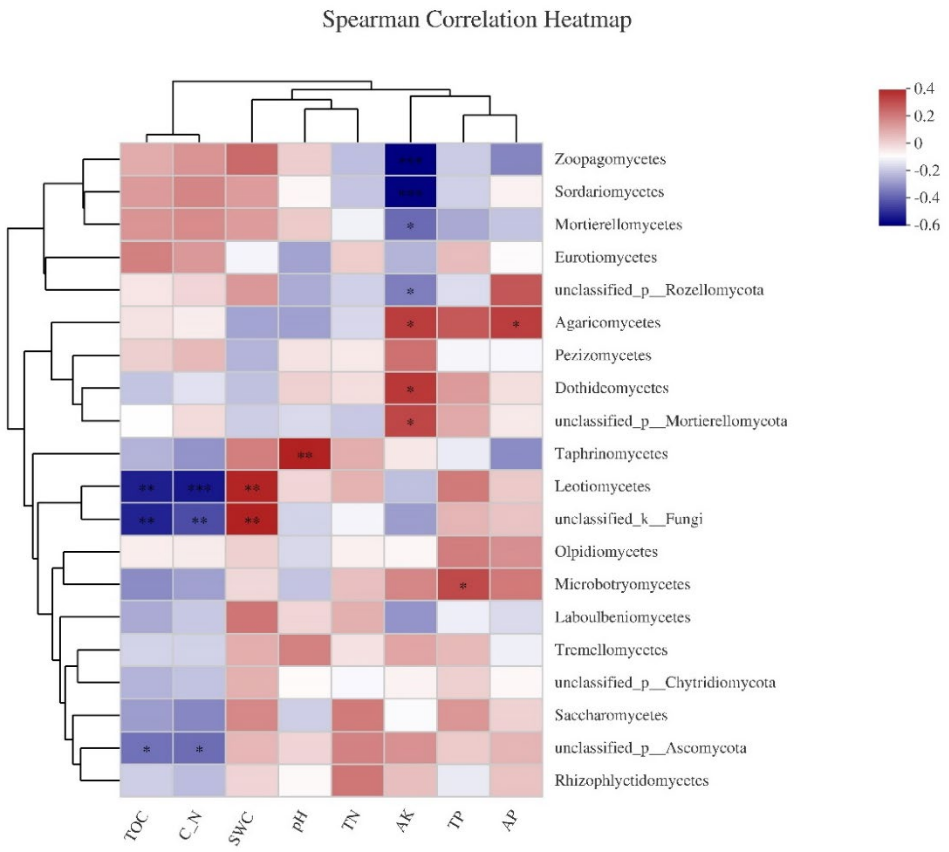

Figure S6. Heatmap of correlation analysis of fungal environmental factors.

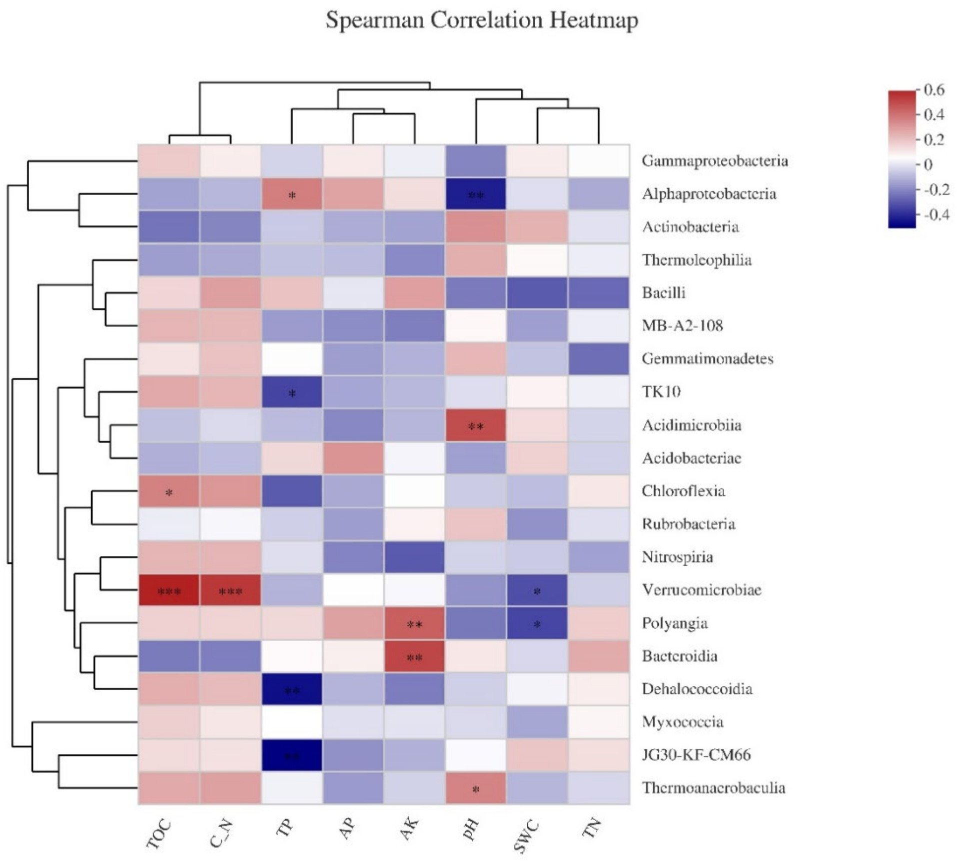

Figure S7. Heatmap of correlation analysis of bacterial environmental factors.

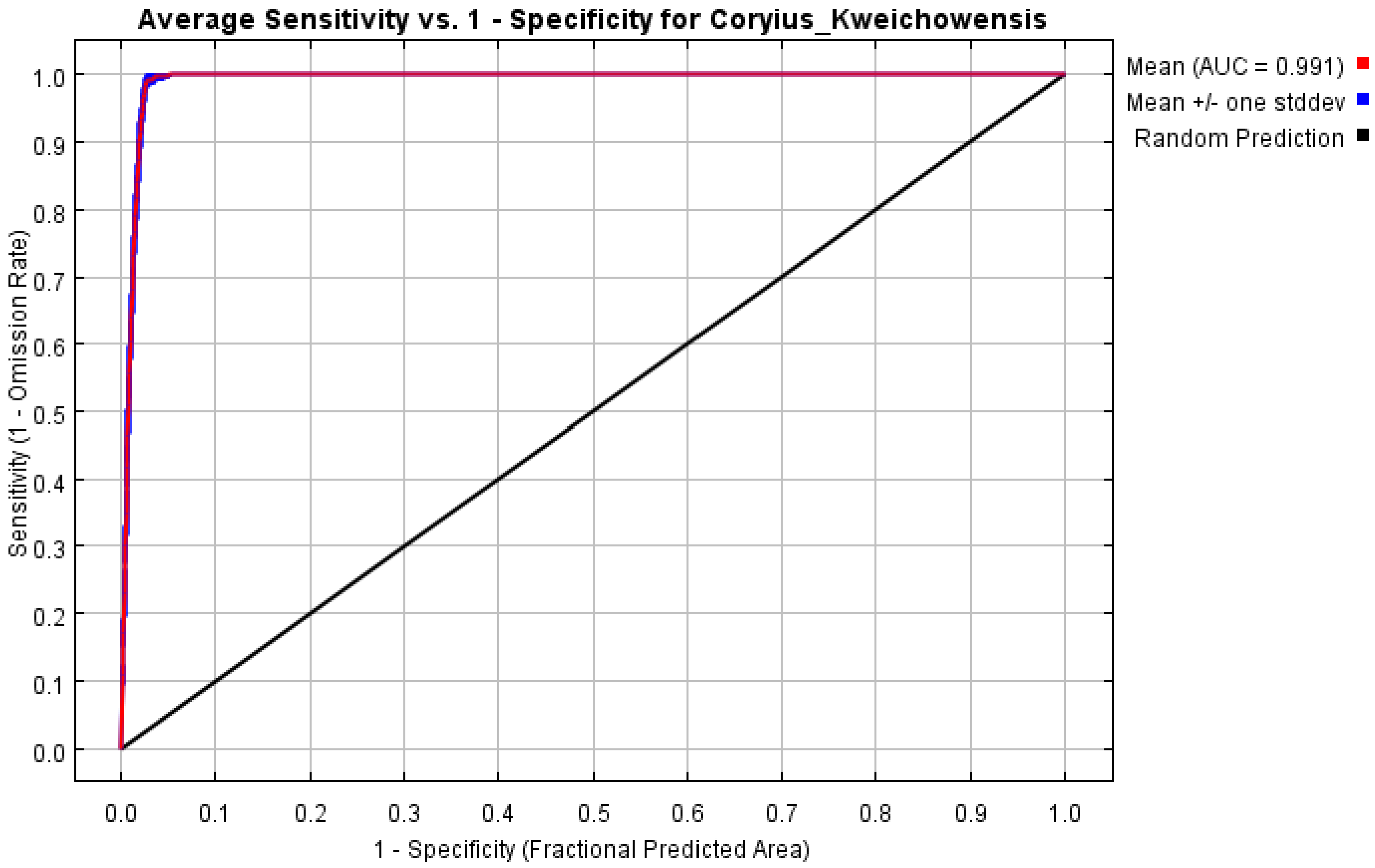

Figure S8. Receiver operator characteristic (ROC) curves of Maxent model.

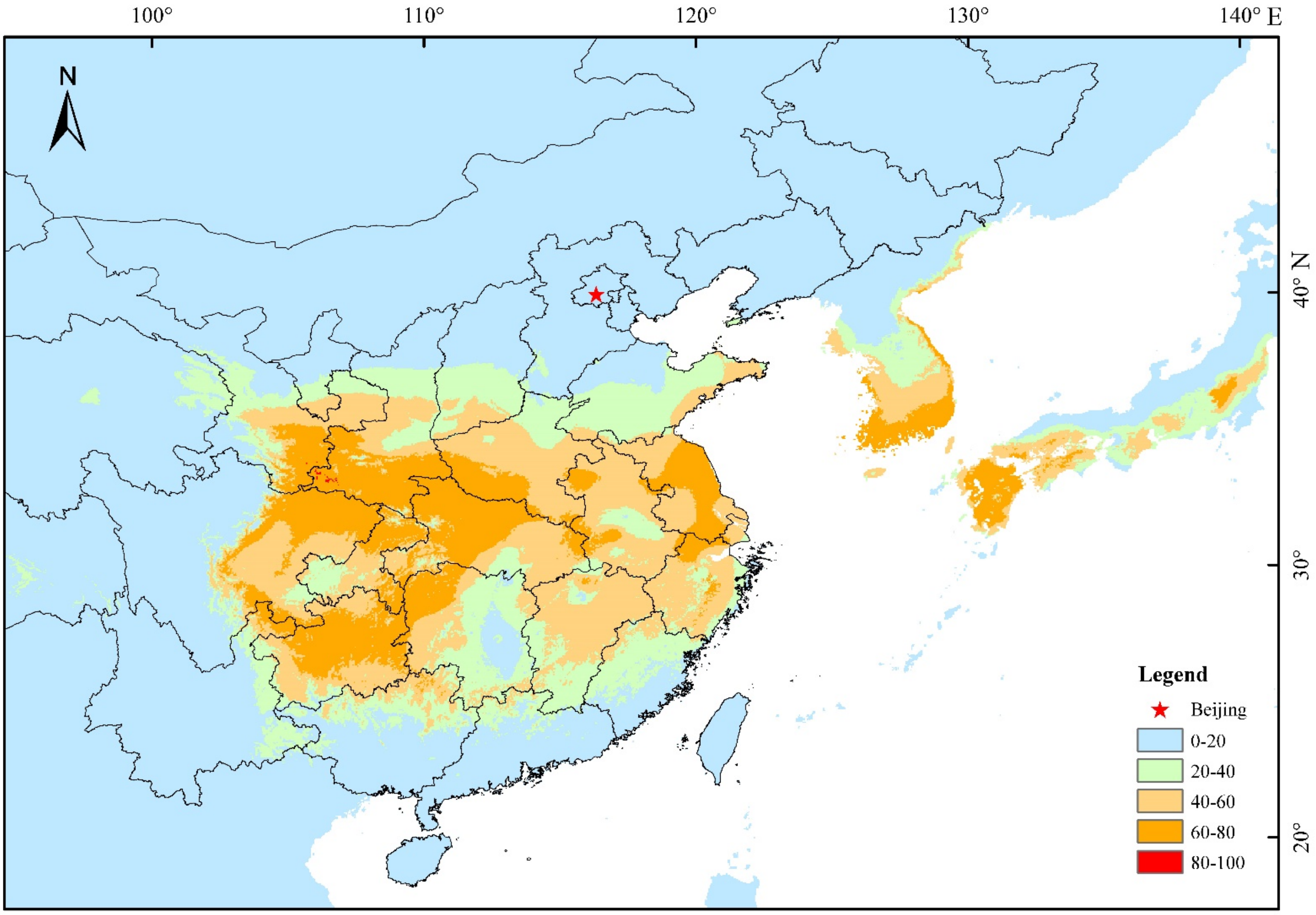

Figure S9. Modelled climatically suitable areas for the C. kweichowensis at 2041–2060.
