# Supplementary figures and images for: Seasonal Changes in Soil Microbial Community and Co-Occurrence Network of Species of the Genus Corylus
Source: Microorganisms. 2021 Oct 26;9(11):2228. doi: 10.3390/microorganisms9112228 (PMC8625130; doi:10.3390/microorganisms9112228)

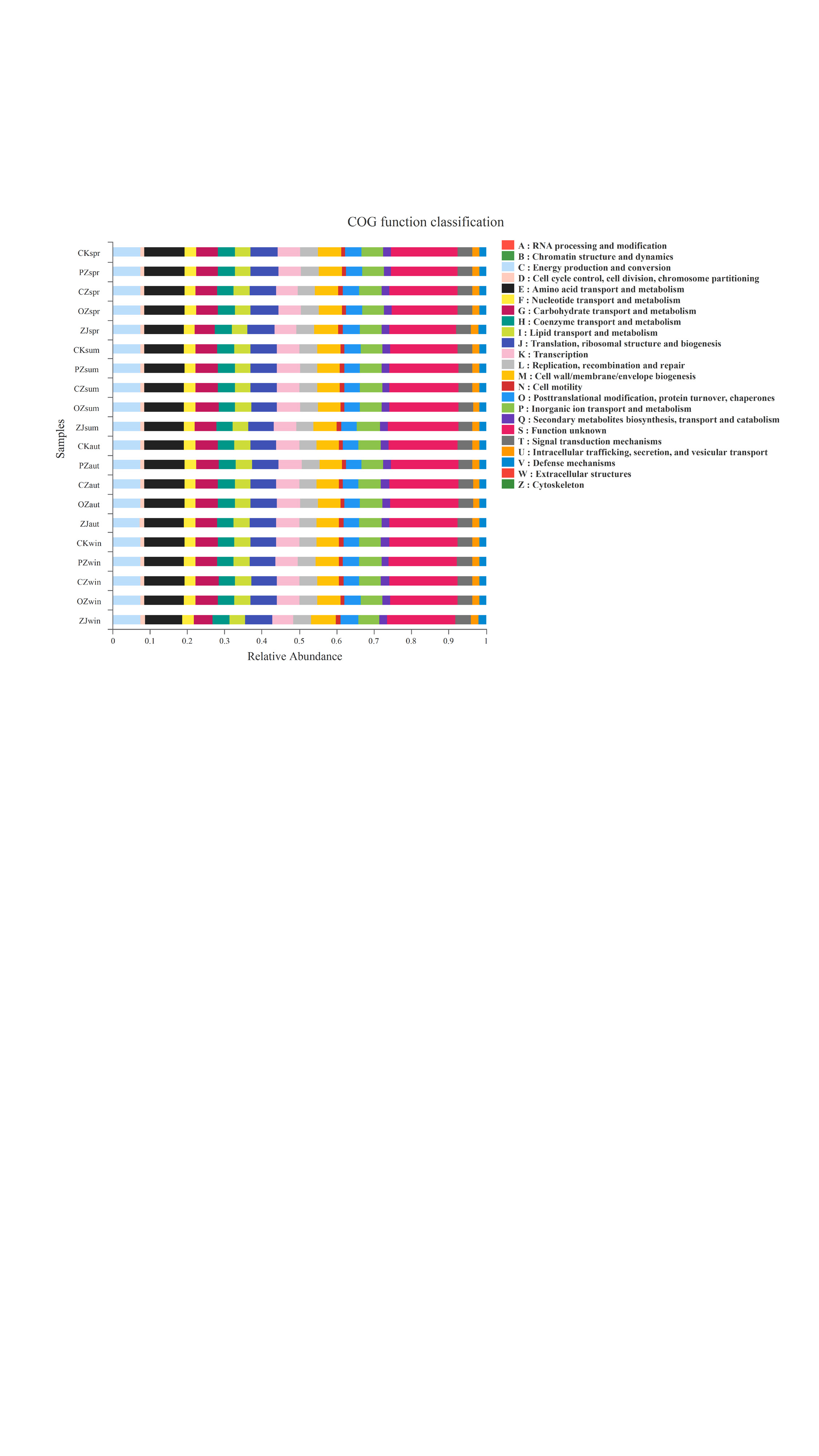

Supplement: Supplementary file 1 [file microorganisms-09-02228-s001.zip › Figure S4.jpg]

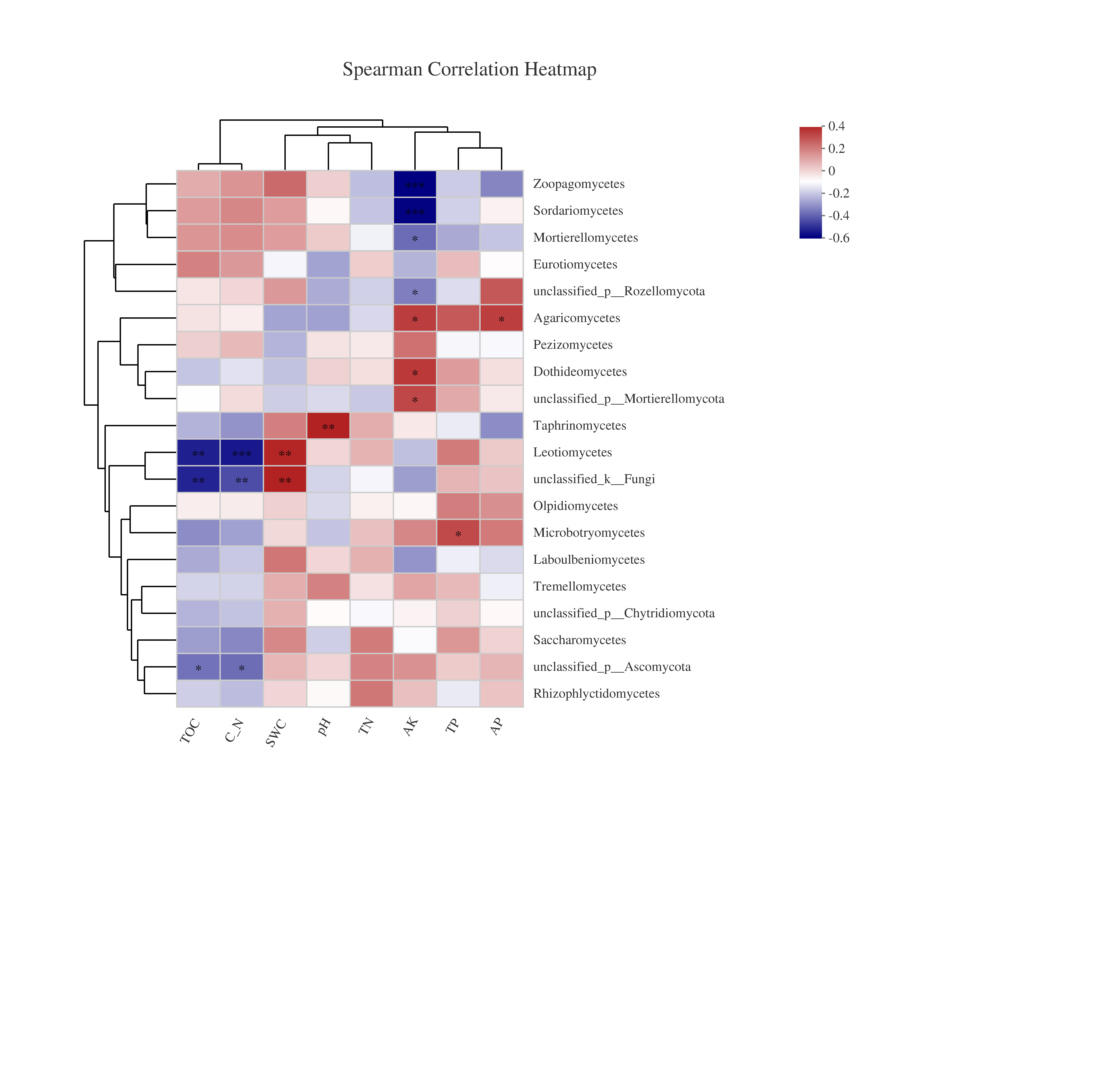

Supplement: Supplementary file 1 [file microorganisms-09-02228-s001.zip › Figure S6.jpg]

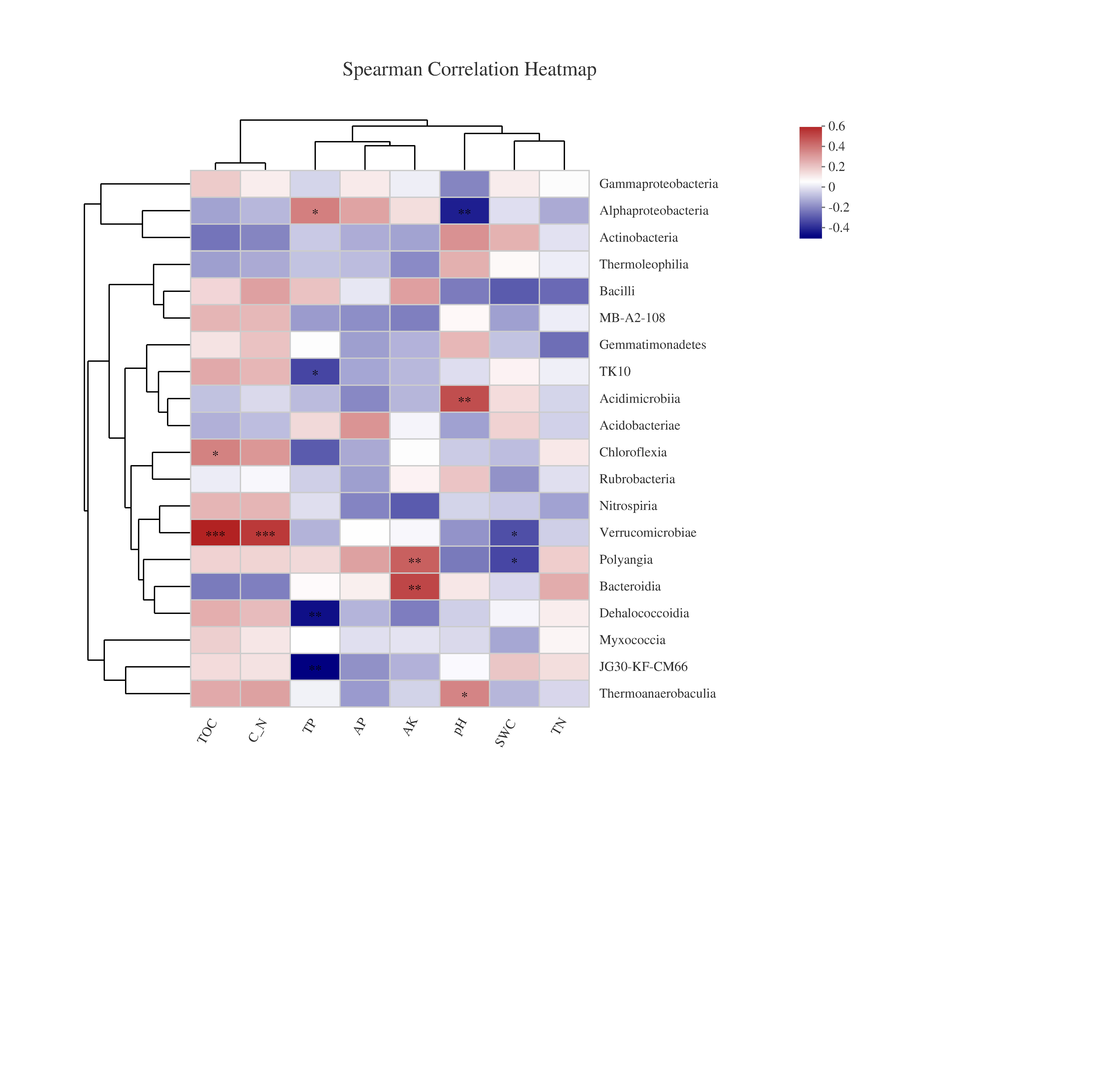

Supplement: Supplementary file 1 [file microorganisms-09-02228-s001.zip › Figure S7.jpg]

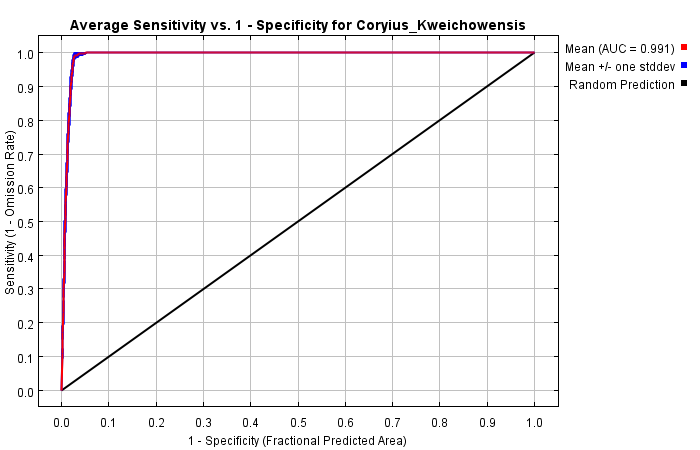

Supplement: Supplementary file 1 [file microorganisms-09-02228-s001.zip › Figure S8.png]

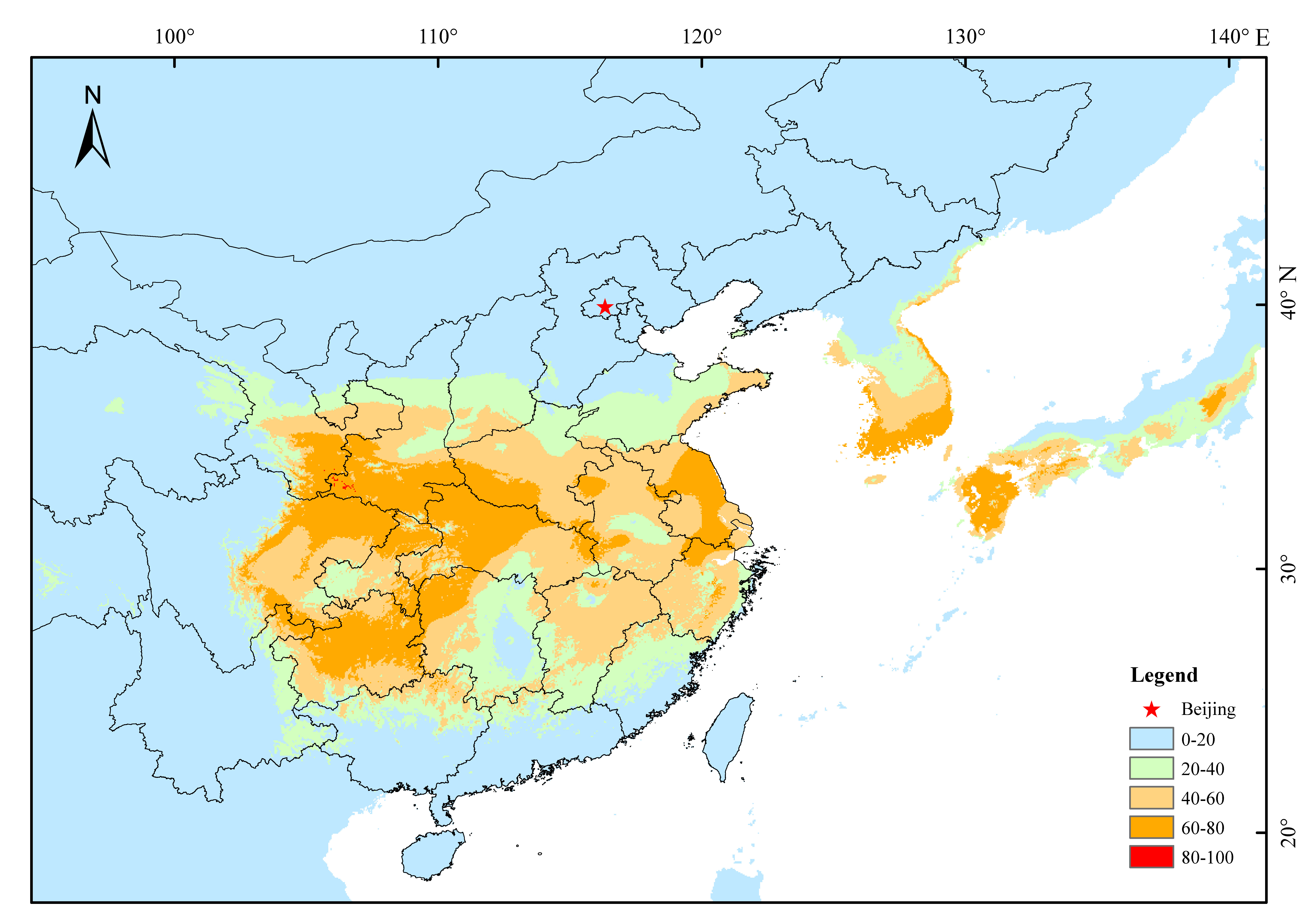

Supplement: Supplementary file 1 [file microorganisms-09-02228-s001.zip › Figure S9.jpg]
